# Comparison of composite and segmental methods for acquiring optical axial length with swept-source optical coherence tomography

**DOI:** 10.1038/s41598-020-61391-7

**Published:** 2020-03-11

**Authors:** So Goto, Naoyuki Maeda, Toru Noda, Kazuhiko Ohnuma, Shizuka Koh, Ikko Iehisa, Kohji Nishida

**Affiliations:** 1 Department of Ophthalmology, Osaka University Graduate School of Medicine, Osaka, Japan; 2 Department of Ophthalmology, National Hospital Organization, Tokyo Medical Center, Tokyo, Japan; 3 School of Optometry and Vision Science, University of California Berkeley, California, 94720 USA; 4 Laboratorio de Lente Verde, Chiba, Japan; 5 Integrated Frontier Research for Medical Science Division, Institute for Open and Transdisciplinary Research Initiatives (OTRI), Osaka University, Osaka, Japan

**Keywords:** Outcomes research, Lens diseases, Optical metrology, Imaging and sensing

## Abstract

This study compared the optical axial length (AL) obtained by composite and segmental methods using swept-source optical coherence tomography (SS-OCT) devices, and demonstrated its effects on the post-operative refractive errors (RE) one month after cataract surgery. Conventional AL measured with the composite method used the mean refractive index. The segmented-AL method used individual refractive indices for each ocular medium. The composite AL (24.52 ± 2.03 mm) was significantly longer (*P* < 0.001) than the segmented AL (24.49 ± 1.97 mm) among a total of 374 eyes of 374 patients. Bland–Altman analysis revealed a negative proportional bias for the differences between composite and segmented ALs. Although there was no significant difference in the RE obtained by the composite and segmental methods (0.42 ± 0.38 D vs 0.41 ± 0.36 D, respectively, *P* = 0.35), subgroup analysis of extremely long eyes implanted with a low power intraocular lens indicated that predicted RE was significantly smaller with the segmental method (0.45 ± 0.86 D) than that with the composite method (0.80 ± 0.86 D, *P* < 0.001). Segmented AL with SS-OCT is more accurate than composite AL in eyes with extremely long AL and can improve post-operative hyperopic shifts in such eyes.

## Introduction

The accurate calculation of intraocular lens (IOL) power is crucial for achieving desirable refractive outcomes after cataract surgery. Post-operative refractive errors are mainly dependent on the following four factors: corneal power, axial length (AL), post-operative IOL position, and IOL quality (i.e. the error from variability in IOL power). Earlier studies have shown that 17–36% of post-operative refractive errors arise from imprecise AL measurements^1,2^. Historically, ultrasound biometry has been the most commonly used technique for measuring AL, anterior chamber depth (ACD), and crystalline lens thickness^3^. The immersion technique is generally considered to be more accurate than the contact technique in ultrasound biometry. However, the development of partial coherence interferometry (PCI) has led to more advanced optical biometry systems, which are ten times more precise than ultrasound for measuring AL^4^. PCI-based systems are now widely used and recognised as the gold standard for measuring ocular biometric parameters^4–7^.

It should be noted that optical and acoustic ALs are not equivalent, because the retinal pigment epithelium (RPE) is the endpoint of the optical measurements, and the internal limiting membrane (ILM) is the endpoint of the ultrasonic measurements. Moreover, the segmental method for AL determination includes the individual sound velocity of each component and the composite method uses the average sound velocity for all the components. Both methods are available for measuring the AL using ultrasound. On the other hand, the composite method measures AL optically via the mean group refractive index for all the components. Recently, segmented ALs measured by an optical low-coherence reflectometry (OLCR) biometer with a peak wavelength of 820 nm were reported to be longer in short eyes and shorter in long eyes, compared to those obtained by composite ALs^8,9^. Although OLCR and PCI can measure the AL with greater precision than ultrasound and do not require any contact with the eye, the mis-segmentation of cornea or crystalline lens is difficult to detect, because OLCR and PCI provide axial and intraocular distances on an A-scan information without two-dimensional cross-sections of the images.

A second generation anterior segment swept-source optical coherence tomography (AS-OCT) was recently developed^10–12^. This novel, but commercially-available, AS-OCT system has an improved scan rate, depth capacity, and density assessment, allowing for sharper images of the anterior and posterior surfaces of the cornea, crystalline lens, and IOL. As a result, corneal thickness, ACD, aqueous depth (AQD: the distance between the corneal endothelium and the lens surface), and lens thickness can be automatically measured using AS-OCT with good repeatability and reproducibility^10–12^. Moreover, a newly introduced optical biometer that is also based on swept-source optical coherence tomography (SS-OCT) has been shown to generate repeatable and reproducible measurements^13–16^.

The current study compared optical ALs obtained with the conventional composite method and the segmental method using SS-OCT based devices and examined the differences in post-operative refractive errors following cataract surgery.

## Results

A total of 374 eyes of 374 patients (251 women [67.1%]) were included in this study. Patients had a mean age of 76.1 ± 8.3 years (range: 40–96 years). All eyes had successful phacoemulsification with IOL implantation. AcrySof Toric and AN6MA IOLs were implanted in 358 and 16 eyes, respectively.

Table 1 summarises the ocular dimensions including the corneal thickness, ACD, AQD, lens thickness, and vitreous length measured with SS-OCT and AS-OCT. These values were significantly different when measured with the two instruments (*P* < 0.001). The intraclass correlation coefficient was 0.992 for the corneal thickness, 0.999 for AQD, and 0.998 for the lens thickness, indicating good agreement for these parameters.

**Table 1:** Comparison of optical biometry measurements acquired with a swept-source optical coherence tomography-based biometer and swept-source anterior segment optical coherence tomography (n = 374 eyes).

	SS-OCT-based biometer			AS-OCT					
	Mean	Min	Max	Mean	Min	Max	Difference	R	P
Corneal thickness (um)	530 ± 31	418	611	544 ± 30	467	625	−13.6 ± 9.06	0.96	<0.001
Anterior chamber depth (mm)	3.15 ± 0.40	2.16	4.23	3.25 ± 0.42	1.96	4.25	−0.10 ± 0.12	0.96	<0.001
Aqueous depth (mm)	2.62 ± 0.40	1.62	3.71	2.70 ± 0.42	1.35	3.71	−0.08 ± 0.12	0.96	<0.001
Lens thickness (mm)	4.63 ± 0.40	3.33	5.79	4.65 ± 0.39	3.4	5.83	−0.02 ± 0.12	0.96	<0.001
Vitreous length (mm)	16.74 ± 1.91	13.44	24.78	16.89 ± 1.83	13.69	24.60	−0.15 ± 0.12	1.00	<0.001

Data are presented as the mean standard deviation as applicable. SS-OCT = swept-source optical coherence tomography, AS-OCT = anterior segment optical coherence tomography, Min = minimum, Max = maximum, R = correlation coefficient.

The mean composite AL_RPE_ was 24.81 ± 1.94 mm (range: 21.52 to 32.93 mm) and the mean segmented AL_RPE_ was 24.79 ± 1.97 mm (range: 21.42 to 32.99 mm). These values were significantly different (*P* < 0.001), but there was a significant correlation between the two values (R = 1.00). The mean composite AL_ILM_ was 24.52 ± 2.03 mm (range: 21.09 to 33.01 mm) and the mean segmented AL_ILM_ was 24.49 ± 1.97 mm (range: 21.12 to 32.69 mm). These values were also significantly correlated (R = 1.00, *P* < 0.001). The mean difference between the composite and segmented AL_ILM_ was 0.038 ± 0.067 mm.

**Figure 1:**
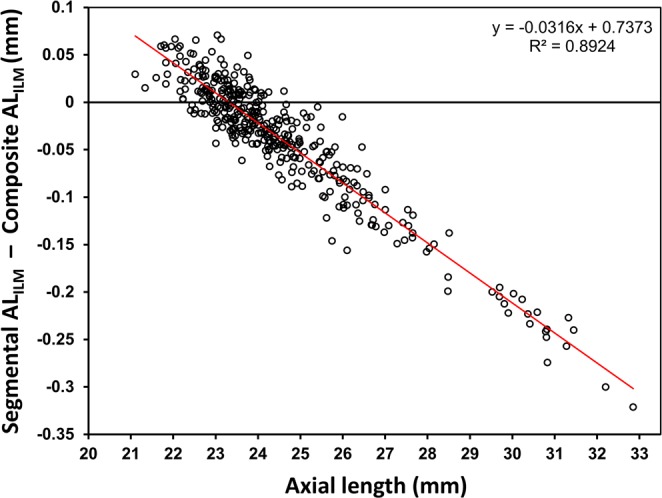
Bland–Altman plots showing the agreement between segmented and composite AL_ILM_ measurements. The solid line indicates the best-fit line (R^2^ = 0.89, P < 0.001). AL = axial length, ILM = inner limiting membrane.

The Bland–Altman analysis revealed a negative proportional bias between the segmented and composite AL, indicating that the difference between segmented and composite AL_ILM_ measurements increased as AL increased (Fig. 1). The segmented AL_ILM_ was up to 0.32 mm shorter in an eye with a composite AL_ILM_ of 33.01 mm which is the longest eye in this study.

The mean absolute refractive error (MAE) was 0.42 ± 0.38 D using the composite AL_ILM_, and 0.41 ± 0.36 D using the segmented AL_ILM_. There was no significant difference in the MAE (*P* = 0.35).

A subgroup analysis was performed for the 15 extra-long eyes implanted with low power IOLs (≤+4.00 D). The same model of IOL (AN6MA) were implanted in these 15 eyes. The mean composite AL_ILM_ was 31.02 ± 0.85 mm (range: 29.80 to 33.01 mm) and the mean segmented AL_ILM_ was 30.78 ± 0.82 mm (range: 29.61 to 32.69 mm, Table 2), which was significantly different (*P* < 0.001). The mean difference between composite and segmented AL_ILM_ measurements in extra-long eyes was 0.24 ± 0.03 mm.

**Table 2:** Predicted refractive outcomes using the composite and segmental methods in extremely long (axial length >29 mm) eyes implanted less than 4.0 diopter intraocular lens (n = 15 eyes).

	Composite Method	Segmental method	P
Mean AL_ILM_ (mm)	31.02 ± 0.85	30.78 ± 0.82	<0.001
Minimum axial length (mm)	29.80	29.61	—
Maximum axial length (mm)	33.01	32.69	—
Mean spherical equivalent (D)	0.80 ± 0.86	0.45 ± 0.86	<0.001
Mean absolute error (D)	0.80 ± 0.86	0.57 ± 0.78	<0.001
Median absolute error (D)	0.46	0.23	—
Median absolute error 95% CI	0.32–1.27	0.14–1.00	—
Number and percentage of eyes within prediction error			
±0.25 D	5 (33.3%)	8 (53.3%)	0.18
±0.50 D	8 (53.3%)	10 (66.7%)	0.003
±1.00 D	11 (73.3%)	13 (86.7%)	0.01

Data are presented as the mean standard deviation.

The Haigis formula was used to calculate intraocular lens power. AL = axial length, CI = confidence interval, D = diopters, ILM = inner limiting membrane.

The MAE using the segmented AL_ILM_ was 0.45 ± 0.86 D, which was significantly smaller than the MAE using the composite AL_ILM_ (0.80 ± 0.86 D, *P* < 0.001). Additionally, the percentage of correct refraction predictions within ±0.25 D, ±0.50 D, and ±1.00 D were 53.3%, 66.7%, and 86.7%, respectively, with segmented AL_ILM_ measurements and 33.3%, 53.3%, and 73.3%, respectively, with composite AL_ILM_ measurements (Table 2). There were significant differences in the percentage of refractive prediction errors between segmented and composite measurements within ±0.50 D (*P* = 0.003) and ±1.00 D (*P* = 0.01), but not ±0.25 D (*P* = 0.18).

## Discussion

**Figure 2:**
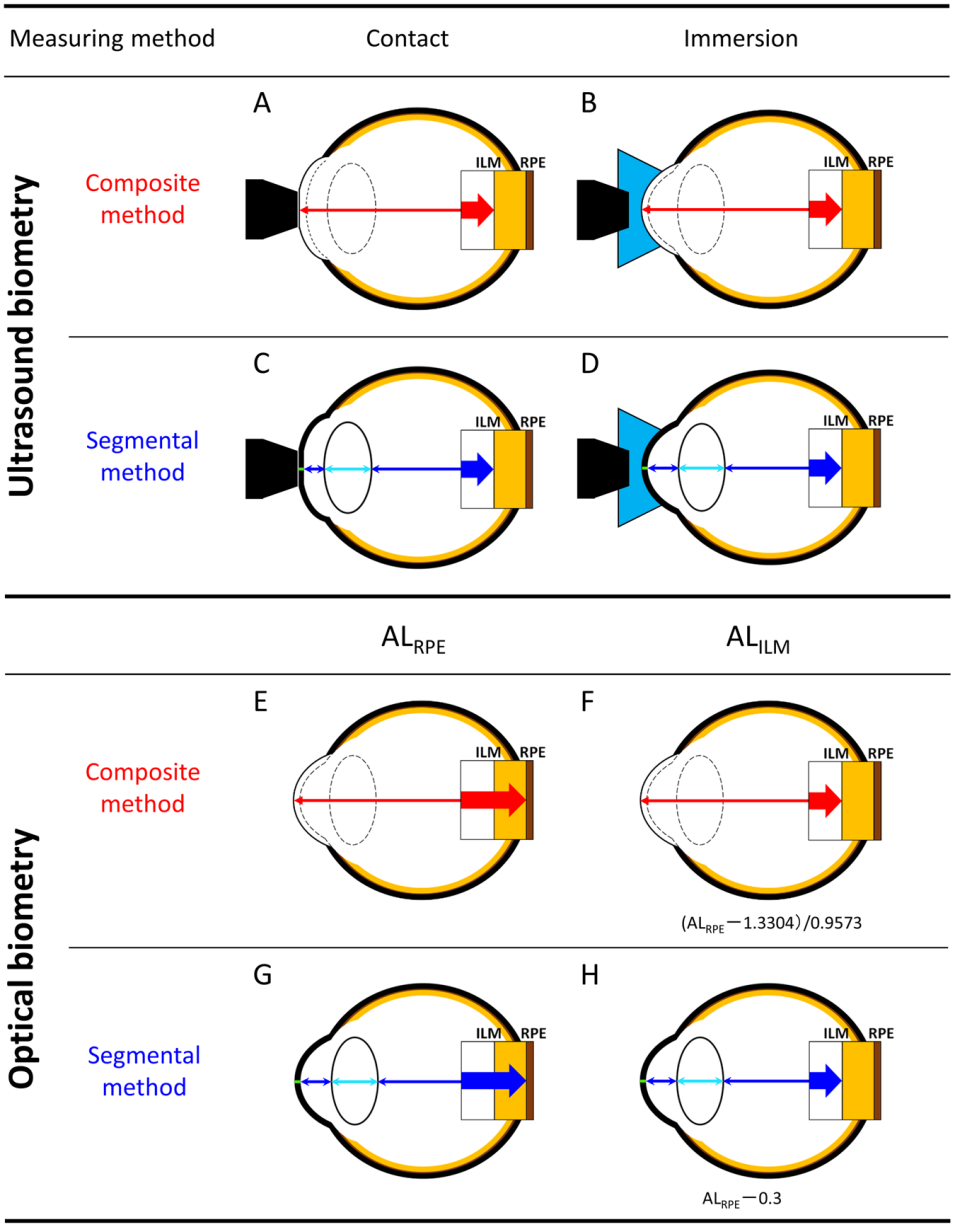
An overview of axial length (AL) measurement strategies for ultrasound and optical AL measurements. (**A**–**D**) Acoustic contact and immersion measurements made using mean group (composite; **A,B**) and individual tissue (segmental; **C,D**) sound velocity. Optical AL measurements using the composite (**E,F**) and segmental (**G,H**) methods. Optical systems (**E,G**) measure the linear distance between the corneal surface and the retinal pigment epithelium (RPE, AL_RPE_) and optical systems (**F,H**) measure the linear distance between the corneal surface and the inner limiting membrane (ILM, AL_ILM_). Since intraocular lens power calculation formulas were developed using the AL_ILM_, each segmented AL_RPE_ measurement was converted to an AL_ILM_ by subtracting the retinal thickness (assumed to be 300 µm in all eyes). Optical composite AL_RPE_ measurements were converted to AL_ILM_ measurements using a regression equation: AL_ILM_ = (AL_RPE_ − 1.3304)/0.9573.

The AL can be calculated in a variety of ways, as shown in Fig. 2. These differences arose from changes in technology and measurement compatibility with IOL power calculation formulas. Since the segmental method with the ultrasound immersion technique is more accurate than the composite method with the ultrasound contact technique, we speculated that the optical measurements with the segmental method would be more precise than the conventional ones with the composite method. Therefore, the present study compared the composite and segmental methods of obtaining optical AL_ILM_ measurements. We found that the difference between the segmental and composite methods in optical AL_ILM_ measurements had a negative proportional bias with AL. This is because the regression Eq. (1) was used to adjust the composite AL_RPE_ to the composite AL_ILM_. As the AL_RPE_ coefficient is 1.0446 (calculated by dividing 1.0 by 0.9573), AL_ILM_ was overestimated in the longer eye using Eq. (1). On the other hand, regarding vitreous length, which is often longer in the longer eye (see Supplementary Fig. S1), the calculated segmented AL is longer than the composite AL due to the difference between the reflective index of the vitreous (1.336), and the mean refractive index of the whole eye (1.349). However, this difference is smaller than the effect of the regression Eq. (1). Therefore, the AL_ILM_ was significantly shorter when acquired with the segmental method than with the composite method in highly myopic eyes. The IOL power calculation using the Haigis formula with segmented AL_ILM_ resulted in a smaller post-operative hyperopic shift than that using the composite AL_ILM_ in the subgroup analysis.

Many IOL power calculation formulas were developed before PCI-based optical biometers were invented. Therefore, one needs to rely upon the ultrasound AL_ILM_^4^. The ultrasound AL_ILM_ with the immersion technique is shorter than the optical AL_RPE_ with the composite method; this difference could induce post-operative refraction errors^7^. Therefore, an established conversion algorithm between optical AL_RPE_ measurements with the composite method and immersion ultrasound AL_ILM_ measurements with the segmental method has been used for a long time^7^. This algorithm, however, is subject to limitations such as an inadequate retinal thickness measurement in eyes with longer ALs. For example, an eye with a 30.0 mm AL_ILM_ should have a retinal thickness of 49 μm (achieved composite AL_RPE_ minus composite AL_ILM_), but an eye with a 20.0 mm AL_ILM_ should have a retinal thickness of 476 µm (10-fold difference). This may be one of the reasons why ALs in long and short eyes tend to be less accurate for predicting refractive outcomes following cataract surgery. In previous studies, the segmented ALs measured by an optical biometer with an 820 nm wavelength were longer in short eyes and shorter in long eyes compared to the composite ALs^8,9^. In the current study, the relationship between segmented and composite AL_ILM_ obtained by SS-OCT with a wavelength of 1060 nm was similar to that reported previously^8,9^.

Although the most significant source of error that contributes to post-operative refractive error comes from the AL^17^, errors in predicted post-operative IOL positions have also become a major contributor to post-operative refractive errors after the introduction of laser biometry^1,2,18^. The post-operative IOL position no longer influences post-operative refractive errors when low power IOLs (<+4.0 D) are used; even a 1.0 mm forward movement in a low power IOL position (e.g., from 4.0 mm to 3.0 mm) influences the refractive outcome by less than −0.2 D, as demonstrated in Table 3. However, the high power IOL has a big influence on the refractive outcome. Therefore, we performed a subgroup analysis on patients that received an IOL with a power less than +4.0 D.

**Table 3:** Theoretical change in refractive outcome with a 1-mm anterior shift in intraocular lens position.

Intraocular lens power	Refractive outcome 3-mm position (D)	Refractive outcome 4-mm position (D)	Refractive outcome change (D)
3.0 D	0.00	0.10	−0.10
4.0 D	−0.80	−0.60	−0.20
5.0 D	−1.60	−1.30	−0.30
7.0 D	−3.20	−2.80	−0.40
8.0 D	−4.10	−3.60	−0.50

Refractive outcomes are presented as the spherical equivalent. Theoretical refractive outcomes were calculated using optical design software with the ray-tracing method. All calculations were made assuming an axial length of 30 mm, an anterior corneal curvature radius of 7.8 mm, and a posterior corneal curvature radius of 6.5 mm. D = diopters.

Previous studies have documented inaccuracies of the popular IOL formulas in long eyes^19–25^. Although the Haigis formula generally has better refractive outcomes in long eyes, post-operative hyperopic errors proportional to the AL have been reported^23^.

The current study did not find a significant difference between predicted post-operative refractive errors when the segmental method and composite method measurements of optical AL were examined in all eyes. However, the post-operative hyperopic shift with the Haigis formula was significantly improved in highly myopic eyes when the optical AL_ILM_ acquired with the segmental method was used. Although the simple replacement of the composite AL_ILM_ with the segmented AL_ILM_ in the traditional IOL power calculations would not help because many formulas are optimised for the composite AL_ILM_, recent studies reported that the segmented AL measured via an optical biometer with a peak wavelength of 820 nm improved refractive prediction accuracy for vergence formulas^8,9^.

Our study had several limitations. First, it is a retrospective single centre study and the population in our subgroup study was relatively small. We used two different IOLs; further investigation of segmented AL measurements in a prospective multicentre study with a larger group of patients and with one model is desirable. Second, the fixation status during the measurement is an important factor for AL accuracy. The SS-OCT biometer enables us to obtain an optical B-scan image during AL measurement. Therefore, it is now possible to check the fixation status during AL measurements and to examine complete longitudinal cross-sectional images of the eye^26–29^. Further studies that utilise these SS-OCT capabilities are needed. Third, our study only measured the effect of AL characteristics on post-operative refractive outcomes. However, other factors are known to influence the refractive outcomes in cataract surgery. The corneal power, in particular, should be investigated more carefully because prior studies have shown that standard keratometry overestimates the corneal power^24^. Finally, the mean group refractive index can vary with cataract grade^30^, and adjustments may be needed for cataract types and stages in future studies.

In conclusion, the segmented AL_ILM_ is more accurate than the conventional one measured with the composite method when using SS-OCT, and the segmented AL measurement reduces the post-operative hyperopic shift in eyes with extra-long AL.

## Methods

### Study participants

This was a retrospective, consecutive case series of all patients who had undergone uncomplicated cataract surgery at a single centre, the National Hospital Organization, Tokyo Medical Center between October 2015 and January 2018. The protocol was reviewed and approved by the Institutional Review Board of the National Hospital Organization, Tokyo Medical Center, and was designed in accordance with the tenets of the Declaration of Helsinki. Informed consent was obtained from all patients. The selection criteria of this study followed the recommendations of a recent editorial by Hoffer *et al*.^31^ regarding best practices for studies of IOL formulas: the use of optical biometry and the inclusion of only 1 eye from each study subject. If patients underwent bilateral cataract surgery, then a randomly selected eye was chosen for inclusion in the study. The exclusion criteria were a best-corrected distance visual acuity after cataract surgery worse than 20/40, a history of ocular surgery, a history of ocular trauma, the presence of a significant ocular comorbidity, unreliable or undetectable preoperative biometry measurements, or a history of intra- or post-operative complications.

All patients underwent cataract surgery through a 2.2-mm corneal incision. One of the following IOLs was implanted: AcrySof Toric (SN6A T3-T6, Alcon, Fort Worth, TX) or AN6MA (KOWA, Nagoya, Japan). All surgical procedures were performed under topical anaesthesia by the same experienced surgeon (TN) and all IOLs were successfully inserted into the capsular bag after phacoemulsification.

### Data collection and patient examinations

All data were retrospectively collected from patient medical records. All patients had undergone preoperative and 1-month post-operative ophthalmic examinations, including a slit-lamp examination, keratometry, and fundoscopy. SS-OCT based biometer (OA-2000; TOMEY CORPORATION, Nagoya, Japan) and AS-OCT (CASIA2; TOMEY CORPORATION) were performed preoperatively. Distant corrected visual acuity, refraction, and intraocular pressure were also measured only by experienced technicians.

#### Swept-source optical coherence tomography based biometer

The SS-OCT based biometer was used to measure AL and corneal power, along with corneal thickness, ACD, and lens thickness using the swept-source laser (1060 nm wavelength). The biometer obtained 10 consecutive scans and automatically calculated the average value.

#### Anterior segment optical coherence tomography

The angle analysis mode in CASIA2 was used to obtain anterior segment images comprising 16 consecutive meridional scans. This instrument uses a super-luminescent diode light source (1310 nm wavelength) and has a scan speed of 50,000 A-scans/second. All OCT images were obtained while the pupil was dilated (topical 0.5% tropicamide and 0.5% phenylephrine hydrochloride). All eyes were imaged in a dark room (illuminance of 0.3 lx) using internal fixation and were obtained twice by a trained technician who was masked to the clinical data.

**Figure 3:**
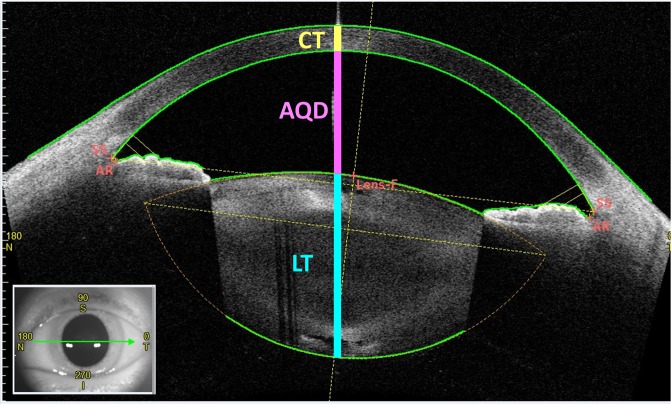
An anterior segment optical coherence tomography (OCT) image showing optical biometer measurements. Measurements were made before cataract surgery along the vertex normal. The inset shows an external photograph with the location and direction of the OCT scan (green arrow). CT = corneal thickness, AQD = aqueous depth, LT = crystalline lens thickness, Lens-f = lens fornix, AR = angle recess, SS = scleral spur, N = nasal, S = superior, T = temporal, I = inferior.

Only images taken with a horizontal (180°) alignment were used in the analysis. Images were centred on the corneal vertex, which was defined as the cross point of the vertex normal and anterior corneal surface. The AS-OCT parameters measured along the vertex normal included corneal thickness, ACD, AQD, and lens thickness. Corneal thickness was defined as the distance between the anterior and posterior corneal surfaces, ACD is the distance between the anterior corneal and anterior lens surfaces, AQD is the distance between the posterior corneal and anterior lens surfaces, and lens thickness is the distance between the anterior and posterior lens surfaces (Fig. 3). The corneal thickness, AQD, and lens thickness were measured by two independent examiners (SG, II) who were masked to the clinical data.

#### Definition of axial length

Figure 2 illustrates how AL was obtained using acoustic and optical measurements. To measure AL, systems used either the composite method, which utilises the mean group index, or the segmental method, which accounts for individual tissue indices of the cornea, aqueous, lens, and vitreous. Ultrasound biometry can measure AL in the following four ways: acoustic contact measurements using mean group sound velocity (Fig. 2A), acoustic immersion measurements using mean group sound velocity (Fig. 2B), acoustic segmented contact measurements using individual sound velocities (Fig. 2C), and acoustic segmented immersion measurements using individual sound velocities (Fig. 2D). Segmented immersion is theoretically the most accurate acoustic measurement because the ultrasound probe does not directly contact the cornea (Fig. 2D).

On the other hand, the SS-OCT based optical biometer measures the optical path length of the whole eye. Currently, it is essential for the optical AL measurement to determine AL_ILM_ from the AL_RPE_, as popular IOL power formulas do not use AL_RPE_ but use AL_ILM_ measurements instead. To estimate composite distance measurements between the cornea and RPE (AL_RPE_, Fig. 2E), the SS-OCT based biometer system uses a mean group refractive index of 1.3496. Then, the following equation (Eq. 1), which is publicly available through the manufacturer, was used to translate AL_RPE_ into composite distance measurements between the cornea and ILM (AL_ILM,_ Fig. 2F) in the current study:

1 $${\rm{Composite}}\,{{\rm{AL}}}_{{\rm{ILM}}}=({\rm{Composite}}\,{{\rm{AL}}}_{{\rm{RPE}}}-1.3304)/0.9573$$

The segmented AL_RPE_ is measured as the sum of the thickness of the cornea, aqueous, lens, and vitreous (Fig. 2G). The refractive indices of the cornea, aqueous, lens, and vitreous were set to 1.376, 1.336, 1.410, and 1.336, respectively^26,32^. Refractive indices of the individual tissue were used during the calculation. The corneal thickness, AQD, and lens thickness were directly measured using AS-OCT. The optical vitreous length was calculated by subtracting the optical path length of the corneal thickness, AQD, and the lens thickness obtained by AS-OCT from the optical path length of the whole eye obtained using an SS-OCT based biometer. Finally, the optical vitreous length was divided by the refractive index of vitreous (1.336) to obtain the vitreous length. The segmented AL_ILM_ (Fig. 2H) was then calculated by subtracting the retinal thickness (previously established as 300 μm^33–35^) from the segmented AL_RPE_.

### Formula calculations

The Haigis formula^7^ was calculated using Excel spreadsheets (Microsoft Corporation, Redmond, WA, USA), and was checked against licensed commercial software on the SS-OCT based biometer. The optimised lens constants from the User Group for Laser Interference Biometry (ULIB) were used for calculations (AcrySof Toric: a0 = 1.78, a1 = 0.40, and a2 = 0.10 and AN6MA: a0 = 1.57, a1 = 0.40, and a2 = 0.10)^36^.

### Data analysis

Composite and segmented AL_ILM_ measurements were compared by using the paired t-test. Bland–Altman plots were used to assess the agreement between the composite and segmented AL_ILM_. The mean post-operative refractive errors (post-operative spherical equivalent minus the predicted post-operative spherical equivalent), mean absolute prediction error (MAE), median absolute prediction error, and standard deviation of the prediction error were calculated with the composite AL_ILM_ and the segmented AL_ILM_. The Wilcoxon test was used to compare the MAE values_._ Subgroup analysis was performed on eyes in which low power IOL less than +4.0 D were implanted. The percentages of eyes with arithmetic prediction errors within ±0.25 D, ±0.50 D, and ±1.0 D were compared between the composite AL_ILM_ and the segmented AL_ILM_ using the McNemar test. Statistical significance was defined as P < 0.05. All statistical analyses were performed using JMP Pro statistical software (version 10.0.2, SAS Institute Inc., Cary, NC, USA).

## Supplementary information

Supplementary Information.

## Data Availability

The datasets generated during and/or analysed during the current study are available from the corresponding author on reasonable request.
