# Supplementary material for: Comparison of composite and segmental methods for acquiring optical axial length with swept-source optical coherence tomography
Source: Sci Rep. 2020 Mar 11;10:4474. doi: 10.1038/s41598-020-61391-7 (PMC7066125; doi:10.1038/s41598-020-61391-7)

**Supplementary Information:**

**Comparison of composite and segmental methods for acquiring optical axial length with swept-source optical coherence tomography**

So Goto^1,2,3^, Naoyuki Maeda^1*^, Toru Noda^2^, Kazuhiko Ohnuma^4^, Shizuka Koh^1^, Ikko Iehisa^2^, Kohji Nishida^1,5^

^1^Department of Ophthalmology, Osaka University Graduate School of Medicine, Osaka, Japan.

^2^Department of Ophthalmology, National Hospital Organization, Tokyo Medical Center, Tokyo, Japan

^3^ School of Optometry, University of California Berkeley, California, 94720, USA

^4^Laboratorio de Lente Verde, Chiba, Japan

^5^Integrated Frontier Research for Medical Science Division, Institute for Open and Transdisciplinary Research Initiatives (OTRI), Osaka University, Osaka, Japan.

***Corresponding author:** Naoyuki Maeda

Department of Ophthalmology, Osaka University Graduate School of Medicine

Room E7, Yamadaoka 2-2, Suita, Osaka, 565-0871, Japan

Tel: [+81-6-6879-3456](tel:%2B81-6-6879-3456)

Fax: +81-6-6879-3458

Email: nmaeda@ophthal.med.osaka-u.ac.jp

**Supplementary Fig. S1**. Scatterplot displaying the correlation between the segmented AL_ILM_ and several ocular parameters; corneal thickness (A), aqueous depth (B), crystalline lens thickness (C), and vitreous length (D) (n = 374). AL = axial length, ILM = inner limiting membrane, r = Pearson's correlation coefficient.


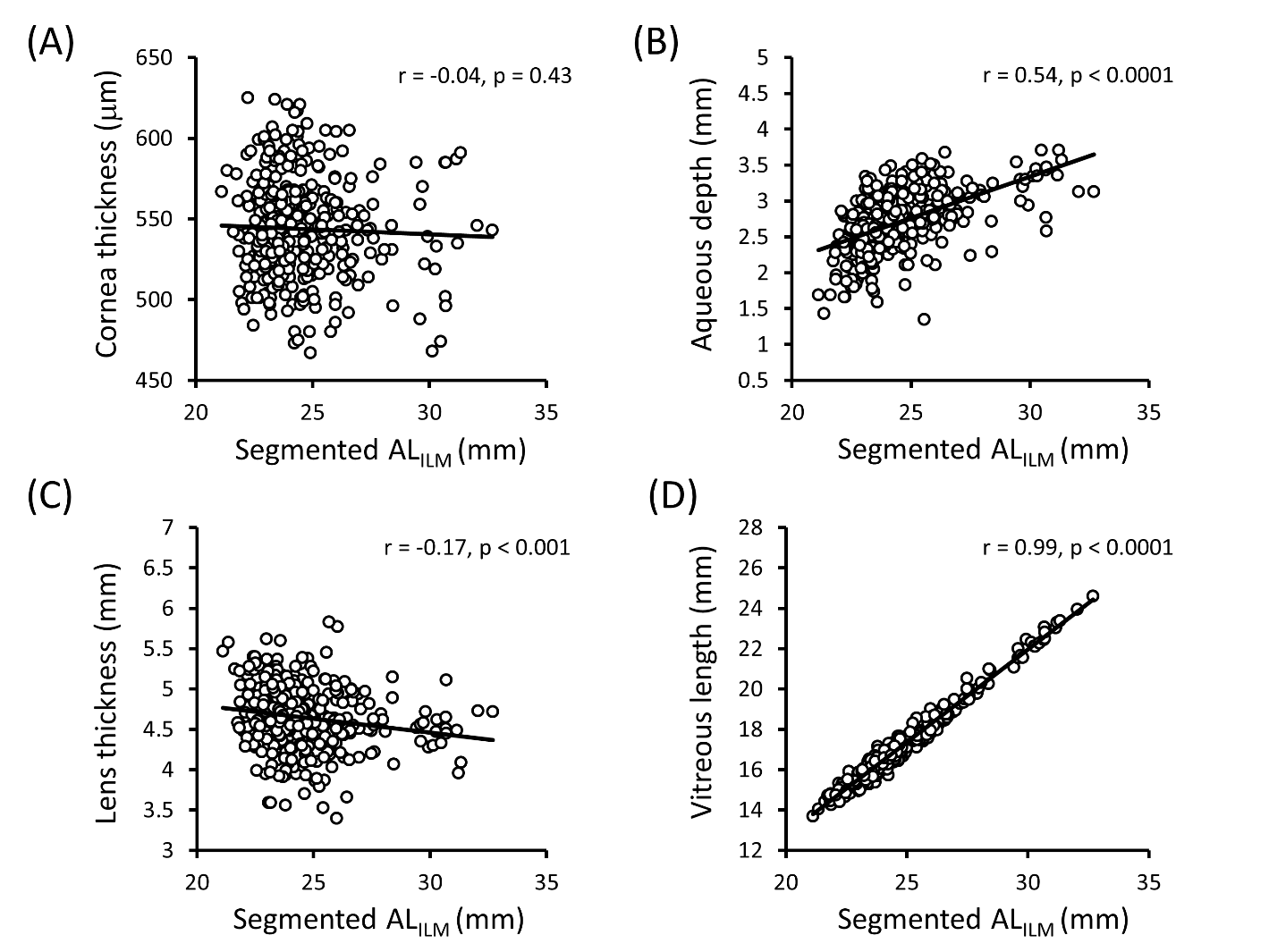

Supplement: Supplementary file 1 — Supplementary Information. [file 41598_2020_61391_MOESM1_ESM.docx]
